# Testing a Personalised Dysautonomia Management Protocol in Patients with Orthostatic Intolerance and a Diagnosis of Myalgic Encephalomyelitis/Chronic Fatigue Syndrome or Long COVID

**DOI:** 10.3390/jcm15072510

**Published:** 2026-03-25

**Authors:** Julia Barr, Lowri Marsden, Theshan Dassanayake, Norah Almutairi, Vikki McKeever, Tarek Gaber, Rachel Tarrant, Belinda Godfrey, Sharon Witton, Manoj Sivan

**Affiliations:** 1 Leeds Institute of Rheumatic and Musculoskeletal Medicine, University of Leeds, Leeds LS7 4SA, UK; julia.barr2@nhs.net (J.B.); wjvp9060@leeds.ac.uk (T.D.);; 2 Leeds Community Healthcare, Leeds LS11 0DL, UK; rachel.tarrant1@nhs.net (R.T.); b.godfrey1@nhs.net (B.G.);; 3 Department of Rehabilitation Medicine, Leeds Teaching Hospitals NHS Trust, Leeds LS7 4SA, UK; 4 Physical Therapy and Rehabilitation Department, College of Applied Medical Sciences, Majmaah University, Al Majma’ah 11952, Saudi Arabia; 5 Leeds and York Partnership Foundation Trust, Leeds LS7 3JX, UK; vikki.mckeever@nhs.net; 6 Wrightington, Wigan and Leigh NHS Trust, Wrightington WN1 2NN, UK; t.gaber@nhs.net

**Keywords:** postural orthostatic tachycardia syndrome, orthostatic hypotension, autonomic medicine, conservative treatment

## Abstract

**Background/Objectives**: Myalgic Encephalomyelitis/Chronic Fatigue Syndrome (ME/CFS) and Long COVID (LC) are complex multisystem conditions with significant functional disability. Many patients experience symptoms of orthostatic intolerance, which can be captured in some cases as Orthostatic Hypotension (OH) or Postural orthostatic Tachycardia Syndrome (PoTS) on objective testing. Conservative treatments are recommended for first-line symptom management, but there is a lack of efficacy evidence. This study aims to assess the feasibility of an 8-week clinically supervised, personalised Dysautonomia Management Protocol (DMP) in a cohort of ME/CFS and LC patients with subjective and objective evidence of orthostatic intolerance (dysautonomia). **Methods**: ME/CFS and LC patients with objective dysautonomia on the 10 min active Lean Test (LT) were recruited to an 8-week DMP, with interventions introduced cumulatively every two weeks. Interventions included increasing daily fluid intake to 3 litres and salt intake to 10 g, pacing to avoid crashes and calf activation. Baseline and weekly data collection included the LT, Composite Autonomic Symptom Score questionnaire (COMPASS-31) and Yorkshire Rehabilitation Scale (YRS). **Results**: Sixteen participants completed the 8-week program, five discontinued during the program, and one was withdrawn following a severe crash. The COMPASS-31 improved by 7.7 points from week 1 to week 8 (*p* = 0.045), with a medium Cohen’s d effect size of 0.55. For the same period, there was a non-significant (*p* = 0.16) improvement in the YRS symptom severity score by 2 points. Comparing the final two weeks of the program with the first two weeks, mean heart rate during the LT decreased by 4.8 beats per minute (*p* = 0.032), with a medium Cohen’s d effect size of 0.44. Adherence to the interventions was highly variable, with none of the patients able to fully employ all four recommendations. **Conclusions**: The results suggest that targeted conservative interventions could influence autonomic function and symptom reduction. However, the magnitude of change was limited, and statistical significance might not necessarily relate to a clinically significant improvement in symptoms.

## 1. Introduction

Myalgic Encephalomyelitis/Chronic Fatigue Syndrome (ME/CFS) and Long COVID (LC) are complex multisystem conditions with significant functional disability and reduced quality of life. Over one million individuals in the UK have ME/CFS or LC [1].

Dysautonomia refers to an abnormality of function of the autonomic nervous system, which, in good health, works in a balanced and rapid manner to maintain automatic control of many of the body systems [2]. It is a condition that is debilitating and can substantially affect quality of life [3]. The autonomic nervous system uses reflexes within the cardiovascular system to maintain cardiac output [2]. A disturbed autonomic function can cause orthostatic intolerance, which can manifest in either Orthostatic Hypotension (OH), which is dizziness with hypotension on standing, or Postural orthostatic Tachycardia Syndrome (PoTS), which is tachycardia without hypotension on standing [4]. Symptoms of dysautonomia include fatigue, dizziness, palpitations, pain, brain fog, thermoregulatory disturbances, bladder/bowel problems and Post Exertional Symptom Exacerbation (PESE) or ‘crash’, as reported by affected individuals [3,5]. Presyncope is common, and some patients with OH or PoTS occasionally faint [6].

ME/CFS and LC are both associated with dysautonomia [7]. A UK-based study found the prevalence of autonomic dysfunction in ME/CFS patients to be 75% [8]. A study of ME/CFS patients in the Netherlands found that 90% had abnormal cerebral flow reduction during orthostatic stress testing, which was found to be related to orthostatic intolerance symptoms [9]. Globally, 66% of LC patients were found to have evidence of moderate to severe autonomic dysfunction using the COMPASS-31 questionnaire [10]. For LC patients in the UK, using objective measures, the prevalence of dysautonomia was found to be 38% in a single centre [7], and in a national multicentre study, it was found to be 15% [11].

The consensus definition of OH as described by Freeman et al. [12] is a reduction in systolic blood pressure of at least 20 mmHg or diastolic blood pressure of 10 mmHg within 3 min, which may be symptomatic or asymptomatic. Sheldon et al. [6] define PoTS as a clinical syndrome characterised by the following:

1. Frequent symptoms that occur with standing, such as light-headedness, palpitations, tremor, generalised weakness, blurred vision, exercise intolerance, and fatigue;
2. A sustained increase in heart rate of ≥30 beats per minute when moving from a recumbent to a standing position (the term ‘sustained’ refers to at least two consecutive 1-min readings);
3. The absence of orthostatic hypotension.

Sheldon et al. [6] summarise two possible mechanisms for PoTS: firstly, autonomic denervation leading to impaired peripheral venoconstriction, which leads to venous pooling, hypovolaemia, and hyperadrenergic stimulation with raised plasma norepinephrine levels, or, secondly, deconditioning, which may be compounded by anxiety and hypervigilance secondary to the underlying physiological abnormalities. Diagnosis of orthostatic intolerance (dysautonomia) can be made with passive tilt table testing or active stand tests, such as the 10 min active Lean Test (LT) [13,14]. Subjective symptoms can be captured by validated questionnaires, such as COMPASS-31 [15] and the Yorkshire Rehabilitation Scale (YRS) [16].

Patients often encounter difficulty in obtaining medical help for these conditions [6]. With patients potentially facing an average wait of 7 years before their condition is formally recognised [17], actions such as validating the patient’s condition promotes a positive patient–physician relationship [4]. There is little data available about the long-term outcomes for patients with PoTS or outcomes of treatments aimed at relieving symptoms and improving quality of life. There are a few outcome studies reporting no mortality from the condition, with the perception that symptoms eventually improve [5,6].

Conservative treatment includes aiming for 2–3 L of fluids a day and 10–12 g of salt a day, using compression garments, and techniques such as squeezing the buttocks and crossing the legs when standing [3,4,5]. Gentle aerobic exercise programs have also been recommended, as has improving sleep hygiene [6,18,19,20]. When non-pharmacological management is not sufficient to relieve symptoms, medications may be used to relieve symptoms rather than aiming for a specific haemodynamic target [5].

Non-pharmacological treatments are recommended as first-line management, but there is a lack of evidence on their efficacy. Most recommendations for treatment are derived from consensus opinions, and consequently, there is a need for good quality data about the efficacy of interventions using standardised reporting tools [3]. Wells et al. [3] also identify that, in the studies that have been performed, patients who have discontinued therapy were excluded from efficacy calculations, meaning that patients for whom the treatment was ineffective or too onerous might have been missed from the analysis.

Driven by the lack of evidence for first-line management using non-pharmacological methods, our research aims to assess the feasibility of using an 8-week clinically supervised, personalised Dysautonomia Management Protocol (DMP) in a cohort of ME/CFS and LC patients with subjective and objective evidence of dysautonomia. The primary objective is to explore the use of the DMP program in terms of changes in the COMPASS-31, YRS scores and 10 min LT, with a secondary objective to assess compliance with advised interventions. This pilot study is the first necessary step before planning larger-scale controlled studies.

## 2. Materials and Methods

### 2.1. Recruitment

**Figure 1:**
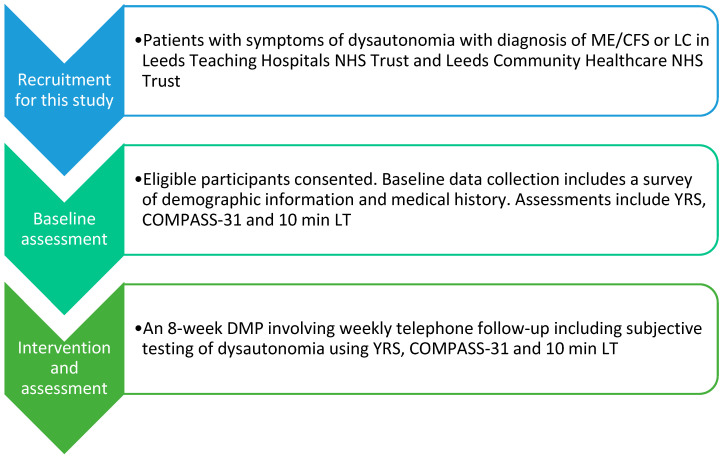
Schematic diagram of study design.

Patients were screened for participation from the Leeds Teaching Hospitals Rheumatology Pain Clinic and the Leeds Community Healthcare Long COVID Rehabilitation service (Figure 1). This study was performed as a service evaluation of the clinics’ normal practice, which was approved by Leeds Teaching Hospitals NHS Trust (Date: 25 November 2024, service evaluation number: SE0335). Informed consent was obtained from participants prior to their recruitment to the service evaluation. Patients were recruited over an eight-month period from 30 November 2024 to 30 July 2025.

The inclusion criteria for this study were the following:

1. Individuals diagnosed with ME/CFS or LC and experiencing symptoms of dysautonomia, such as fatigue, dizziness, palpitations, pain, brain fog, thermoregulatory disturbances, bladder/bowel problems and PESE or ‘crash’ episodes.
2. Evidence of dysautonomia when objectively tested using the 10 min LT.
3. Ability to understand and willingness to sign a written informed consent document.
4. Stated willingness to comply with all study procedures and availability for the duration of the study.
5. Ability to read and understand English.

The exclusion criteria were the following:

1. Pregnancy.
2. Individuals diagnosed with advanced heart disease, e.g., severe heart failure, recent myocardial infarction, uncontrolled hypertension, or ongoing investigations for arrhythmias.
3. Inability to provide informed consent.

### 2.2. Intervention and Follow-Up Cycles

Once the baseline study questionnaires and 10 min LT were completed, participants were initiated on the 8-week clinically supervised DMP. At the end of each full week, participants completed the study questionnaires and a 10 min LT. The DMP was tailored to each patient and was comprised of the following four interventions.

#### 2.2.1. Two Weeks: Increase Fluid Intake to 3 L a Day

Current UK guidelines recommend adults drink 6–8 cups of water a day, which is equivalent to 2 L of water. This includes fluid as part of food, for example soup and yoghurt. We aimed for our participants to increase fluid intake to a total of 3 L per day. This is a moderate increase from the recommended guideline, with the rationale of giving an oral fluid challenge to boost circulating blood volume and improve orthostatic symptoms.

Patients received information during their weekly meeting, including guidance on calculating fluid intake, along with a written information sheet. Fluid intake can be monitored with certain-sized containers, bottles or glasses, and patients choose a method that works for them. The amount of fluid in food was estimated either by how much is added at the start of cooking or by the volume of the container that it comes from. Patients were encouraged to monitor their intake and then increase it to the target amount once they were confident they had identified a monitoring method that suited them, which could include recording it on their phone or on paper. At the meeting following the first week of the intervention, their progress was reviewed, and further advice was given about reaching or modifying the target.

In our cohort of patients without major comorbidities, this intervention was considered low risk. The fluid target was reduced to 2.5 L daily for those with a body weight < 50 kg. With increased fluid intake, there is a risk of fluid overload or electrolyte imbalances. Symptoms could include swelling, rapid weight gain, shortness of breath, headaches, bloating and high blood pressure. Patients were advised to reduce their fluid intake if they were consuming more than 3.5 L daily. In otherwise healthy individuals, the risk of drinking 3 L of fluid a day is very low.

#### 2.2.2. Two Weeks: Increase Salt Intake to 10 g a Day

Current UK guidelines recommend adults consume 5 g of salt a day, which is equivalent to one teaspoon of table salt. We aimed for our participants to increase this to 10 g of salt a day (this is routine practice in the management of dysautonomia), including what they were already consuming in their diet. This is a moderate increase from the recommended guideline, with the rationale of increasing the absorption of water from the gut and retention within the circulating volume for a longer period to boost circulating blood volume and improve orthostatic symptoms.

Patients received information in their weekly meeting about calculating salt intake, alongside a written information sheet. Salt intake can be monitored by noting packaged food labels and monitoring salt added to food. Advice was tailored to their specific circumstances as clinically indicated. Patients were encouraged to monitor their intake and then increase it to the target amount once they were confident they had found a method of monitoring that suited them. At the meeting following the first week of the intervention, their progress was reviewed, and further advice was given about reaching or modifying the target.

In our cohort of patients who lack significant comorbidities, this was also considered a low-risk intervention. In patients with comorbidities, there would be a risk of fluid retention, salt imbalances and hypertension. Symptoms could include bloating, thirst, frequent urination, high blood pressure, swelling, headaches and fatigue. The target was reduced to 5 g a day for one participant who was 63 years old and had pre-existing hypertension.

#### 2.2.3. Two Weeks: Pacing to Avoid Crashes

In this part of the intervention, patients were asked to pace their activities to avoid crashes. Many patients already prioritised their activities and planned their energy expenditure, but if they did not, then this was a focus for these two weeks. If they were already employing these techniques, then they were encouraged to give themselves permission to prioritise their pacing as well as incorporating a period of active rest daily. The way that each participant approached the intervention was personal to them. The specific focus for each participant was discussed at the weekly intervention meeting and was agreed based on what the participant felt was feasible. Principles included pacing to have 20% of their energy left at the end of the day, listening to their body for signs that they need to rest and finding and implementing a method of active rest that worked for them. The rationale is that these activities promote parasympathetic activity and reduce the effects of the sympathetic nervous system. Patients were asked to report on their crashes and fatigue levels.

This intervention was anticipated to affect their social and work lives, as patients may choose to prioritise activities that they are able to achieve without going beyond their energy envelope. It was the patient’s choice as to how to schedule their activities or include active rest to pace their activities.

#### 2.2.4. Two Weeks: Calf Activation Movements

Patients were asked to perform calf activation movements in either a lying, sitting or standing position, depending on their functional abilities. They were asked to perform 10 repetitions of the movements at least 3 times a day, with modifications made to improve compliance based on each patient’s needs. These movements aim to activate the calf muscle pump to return blood to the central circulation, thereby reducing orthostatic symptoms. Patients were warned that they may experience some discomfort in their calf muscles due to the increased activity, and this should settle in the second week as their muscles get used to the increased activation.

The four interventions were tested sequentially and cumulatively for each patient to estimate which had the greatest effect, recognising that there would be a summative effect of the interventions. During all these interventions, patients were monitored weekly in the planned study meetings. Each intervention was introduced with a discussion and an information sheet. Patients were able to contact the study team by email and telephone if advice was needed, and they were also aware that they could stop an intervention should they experience side effects.

Every stage of this study was performed remotely through Teams using an NHS Microsoft Teams (Redmond, WA, USA) account. Participants had weekly Teams meetings to discuss the interventions and to collect outcome measures, including subjective scales of Composite Autonomic Symptom Score (COMPASS-31) and Yorkshire Rehabilitation Scale (YRS) and objective testing of Blood Pressure (BP) and Heart Rate (HR) changes during the 10 min LT.

### 2.3. Outcome Measures

The COMPASS-31 is designed to be a self-administered subjective questionnaire, which is commonly employed for evaluating and measuring the intensity of autonomic dysfunction symptoms in patients [15]. It consists of 31 items divided into six categories, each indicating a specific element of autonomic function. These domains include orthostatic intolerance, vasomotor, secretomotor, gastrointestinal, bladder, and pupillomotor symptoms. The questionnaire assigns a score to each item based on its severity and frequency, resulting in a total score that ranges from 0 to 100. Greater scores suggest a higher degree of autonomic dysfunction [21]. It was adapted for use in this study by reducing the symptom recall period to one week to have an insight into the impact of autonomic symptoms over the course of the interventions.

The YRS is also designed as a self-administered subjective questionnaire, which is validated for assessment and monitoring in LC [16]. It uses a 17-item score including symptom severity (score range 0–30), functional disability (score range 0–15), additional symptoms (score range 0–25) and overall health (score range 0–10) to capture persistent symptoms and disability in LC. Individuals score their symptoms on a four-point scale ranging from no problem to a severe problem affecting all aspects of daily life. A higher score denotes greater symptom severity and functional disability.

The 10 min LT is a clinical test performed to evaluate orthostatic intolerance, specifically in the context of investigating conditions like PoTS and OH [22]. The test begins with the participant lying supine. Heart rate and blood pressure are measured at 3 min and 5 min of resting supine to establish baseline values; if these values were not within 10 units of each other, the measurement was repeated until the parameters had settled. Following that, the participant was asked to stand up and lean against a wall while keeping heels about 20 cm away from it whilst the back and elbows touch the wall. While leaning, heart rate and blood pressure are obtained immediately after standing and every minute thereafter for 10 min. A positive 10 min Lean Test (LT) is a significant increase in heart rate upon standing by more than 30 beats per minute (or more than 40 beats per minute in 12–19 year olds) without a substantial drop in blood pressure (which is diagnostic for PoTS) or a significant drop in systolic blood pressure by more than 20 mmHg or diastolic blood pressure by more than 10 mmHg within the first 3 min (which is diagnostic for OH). Patients were supplied with an Omron (Kyoto, Japan) M2 blood pressure machine with the appropriately sized BP cuff. In order to standardise patient self-measurements, the first 10 min LT was performed during a Teams meeting and observed for correct technique. Only symptoms were discussed during the tests, with no other conversation in order not to influence results.

The COMPASS-31 questionnaire, YRS questionnaire and 10 min LT template can be accessed in the Supplementary Material document.

### 2.4. Statistical Analysis

The scores for the subjective outcome measures of the COMPASS-31 and YRS were analysed using a student’s paired two-tailed *t*-test. Statistical analysis for the objective outcomes of the weekly 10 min LT was also performed using a student’s paired two-tailed *t*-test. To rule out the risk of type I error due to the longitudinal nature of the data, positive findings were also analysed using a repeated-measures ANOVA.

## 3. Results

**Figure 2:**
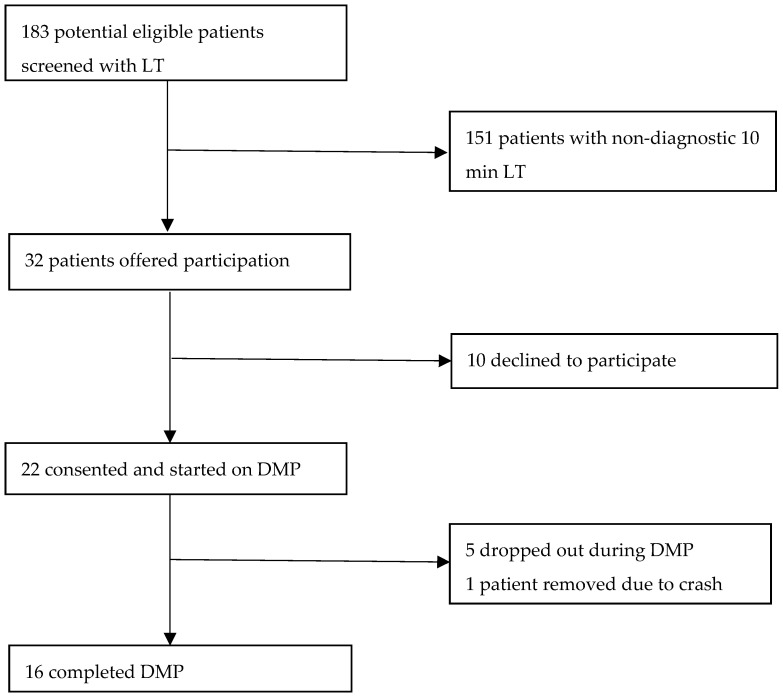
Recruitment flow chart.

As detailed in Figure 2, a total of 183 patients with a diagnosis of ME/CFS or LC underwent screening with the 10 min LT. Of these, 151 yielded non-diagnostic results (for OH or PoTS) and were deemed ineligible. There were 32 patients who met the inclusion criteria and were invited to participate; a total of 22 consented and initiated the DMP. Finally, 16 participants completed the 8-week program, five discontinued during the program, and one was withdrawn following a severe crash related to a surgical procedure.

Among the 22 recruited participants, 63.6% had an ME/CFS diagnosis, with the remaining 36.4% having an LC diagnosis. The mean age was 39 years (range 17.25–63.2 years), and 90.9% were female. By ethnicity, 18 were White British, two were Mixed, one was Arab, and one was White Other. The mean Body Mass Index (BMI) was 29.2 kg/m^2^ (range 17.7–47.9 kg/m^2^). The mean duration of dysautonomia symptoms was seven years (range, 0.6–30 years). On the baseline 10 min LT, 20 patients (90.9%) met the criteria for PoTS, and two patients (9.1%) met the criteria for OH. The baseline characteristics for the 22 consented and for the 16 patients who completed the DMP are detailed in Table 1.

All the 22 consented patients reported comorbidities, as detailed in Table 2. There were 10 patients who reported also having a diagnosis of fibromyalgia, seven had asthma, and Ehlers–Danlos Syndrome (EDS) and hypermobility syndrome were reported by six patients. Each of the following conditions was reported by four patients: depression and/or anxiety, chronic pain, migraine and reflux. Irritable bowel syndrome was reported by three patients. Raynaud’s phenomenon, ADHD or autism was reported by two patients. In addition, each of the following conditions was only reported by a single individual: type 2 diabetes, hypertension, fatty liver, epilepsy, overactive bladder, endometriosis, sleep apnoea, hyperhidrosis, restless legs, and vertigo.

Table 3 lists the medications the patients were taking. All patients were on at least one medication, with most on multiple medications. The most common class of medications was pain relief medications, including duloxetine, gabapentin, pregabalin, amitriptyline, paracetamol and ibuprofen.

### 3.1. Primary Outcomes

The COMPASS-31 improved by 7.74 points from week 1 to week 8 (*t*-test and repeated-measures ANOVA *p* = 0.045), with a medium Cohen’s d effect size of 0.55 (Table 4). No significant changes were detected in YRS domains. Comparing the final two weeks of the program with the first two weeks, mean heart rate during orthostatic testing decreased by 4.8 beats per minute (*t*-test *p* = 0.032, repeated-measures ANOVA *p* = 0.005), with a medium Cohen’s d effect size of 0.44. No statistically significant changes were observed for systolic or diastolic blood pressure between the beginning and end of the program.

**Figure 3:**
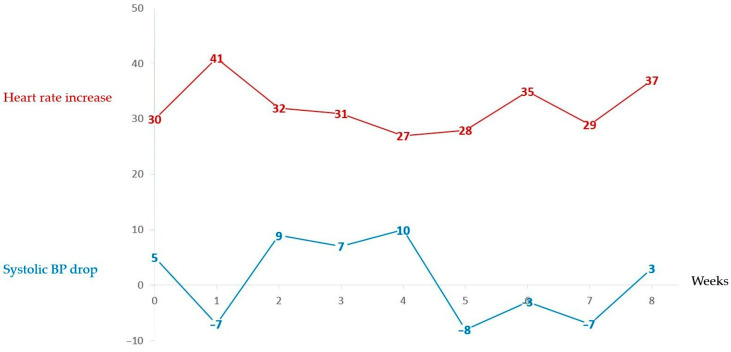
Change in heart rate and drop in systolic blood pressure for an individual over the 8-week DMP who displays a consistent PoTS profile.

**Figure 4:**
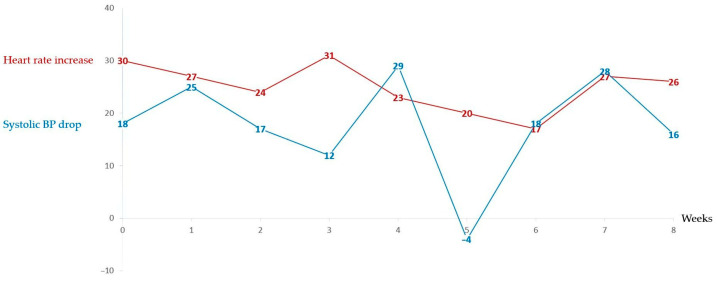
Change in heart rate and drop in systolic blood pressure for an individual over the 8-week DMP who meets the PoTS profile twice, OH profile three times, and, on the other occasions, does not meet the diagnostic criteria for either OH or PoTS.

Serial 10 min LTs demonstrated that most participants exhibited a PoTS phenotype consistently over time, whereas a subset fluctuated in tachycardia magnitude or oscillated between PoTS, OH, and borderline profiles. Figure 3 and Figure 4 demonstrate weekly 10 min LT changes for two individuals, one who consistently responds with a tachycardia but maintains their blood pressure, thus having a consistent PoTS profile, and the second who some weeks has an isolated tachycardic response, some weeks has an orthostatic hypotension and, on other occasions, has borderline changes that do not meet either diagnostic criteria.

Among those with ME/CFS (*n* = 11), four had a consistent PoTS profile across assessments, with others alternating between PoTS and OH (*n* = 4) or PoTS and borderline readings (*n* = 3). In LC (*n* = 5), three showed alternating among PoTS, OH, and borderline profiles, with one each alternating PoTS/OH and PoTS/borderline, as detailed in Table 5. These findings highlight an individual’s variability in dysautonomia profiles and the potential for day-to-day phenotype shifts.

### 3.2. Secondary Outcome

Adherence to the interventions amongst the 16 patients who completed the DMP was highly variable. None of the patients were able to fully employ all four recommendations. Nine (53.3%) achieved their daily fluid target, four (25%) achieved their personalised salt target, nine (56.3%) implemented pacing or active rest, and 10 (62.5%) met the calf-activation movements target. No statistically significant differences were found between participants who achieved one, two or three out of four interventions during the programme.

### 3.3. Adverse Events

No intervention-related serious adverse events were recorded. One participant was withdrawn after a severe symptom exacerbation, which was associated with an unrelated surgical procedure. Five additional participants discontinued participation during the DMP for lack of time in their daily schedule and other unspecified personal reasons.

## 4. Discussion

This pilot study evaluates the feasibility of an eight-week clinically supervised DMP in individuals with ME/CFS or LC who demonstrated objective evidence of orthostatic intolerance (dysautonomia). Our findings indicate that the DMP was associated with modest but statistically significant improvements in autonomic symptom burden and symptom severity, alongside a small reduction in orthostatic heart rate, while blood pressure parameters remained unchanged. These results align with previous reports suggesting that non-pharmacological interventions may confer symptomatic benefit in dysautonomia, albeit with variable adherence and clinically modest effect sizes [3]. On analysis, it was not possible to detect whether the diagnosis (ME/CFS or LC) influenced the response to interventions observed in this study.

The observed 4.8 bpm reduction in postural heart rate change and improvement in COMPASS-31 scores, both with medium effect sizes, suggest that targeted lifestyle interventions could influence autonomic function and symptoms. However, the magnitude of change was limited, and statistical significance might not necessarily relate to a clinically significant improvement in symptoms. There are four previous studies that found that ingesting a 480 mL fluid bolus resulted in reduced heart rate on standing by an order of 15–18 beats per minute [23,24,25,26] and improved cognitive function [26]. These studies were also small, with 7–20 participants in each. The lack of blood pressure change in our study is consistent with prior studies indicating that volume expansion strategies primarily affect heart rate rather than vascular tone in PoTS [12]. In older patients without dysautonomia, a pressor response is seen with a 480 mL fluid bolus, leading to the hypothesis that oral water intake triggers a vasoconstrictor response in addition to increasing blood volume [27]. Two studies show that a high salt intake in patients with PoTS increases the blood plasma volume and reduces adrenaline, resulting in improved symptoms and a reduction in heart rate on standing by an order of 9–13 beats per minute [28,29]. Salt intake has also been correlated with a reduction in migraines, which are common in patients with dysautonomia [30]. These small studies each look at a single intervention and, in the case of the fluid studies, only single boluses and not sustained intervention.

It is clear that the pathophysiology is complicated and that patients with dysautonomia are likely to respond to these interventions, but the true effect size is unknown. There are no established Minimal Clinically Important Difference (MCID) thresholds for either the COMPASS-31 or the 10 min LT to compare our results against. As a pilot feasibility study, the primary goal was to evaluate the potential for meaningful change that could justify investigation in a fully powered trial rather than to prove the intervention’s effectiveness in this instance. The moderate improvements in COMPASS-31 scores and reduction in orthostatic heart rate suggest that the DMP could offer clinically relevant improvements for ME/CFS and LC patients. The complexity of these conditions and the long-term nature of the symptoms need an investigative approach that supports the long-term management for patients with symptomatic orthostatic intolerance.

Adherence varied across interventions and for individuals over the course of the program. Calf activation movements were the most achievable, followed by fluid intake and pacing, while increased salt intake was least well implemented. It is not possible with this small sample of patients to determine whether those who adhered to the protocol interventions the best had the most improvement in their objective or subjective measures. With a larger cohort, these subgroups could be examined in more detail. Difficulties in implementation highlight that, despite expert consensus recommending these strategies, meeting and maintaining them are unachievable, even in a short-term, supervised program.

Importantly, the structured weekly review may have contributed to perceived benefit through enhanced validation and therapeutic engagement, a factor previously recognised as critical in managing chronic dysautonomia syndromes [4,6,8,9,10,11,31]. The intensive interaction from the weekly supervised meetings could itself have influenced subjective outcomes, such as the COMPASS-31 and YRS, and as such, improvements in self-reported symptoms burden may reflect contextual factors rather than physiological change. When recommending conservative interventions, such as those in this program, clinicians should set realistic expectations for patients and recognise that these interventions can be burdensome. It is possible that the program activities and weekly review meetings could have contributed to fatigue for participants. It is likely that, if individuals are given more time to implement changes with lower frequency of follow-up, adherence to the interventions could improve, which might allow patients to pace effectively, thereby making changes more detectable.

Serial 10 min LT testing revealed that, although most patients are consistent with one dysautonomia profile, there are patients who fluctuate between PoTS, OH, and borderline profiles, which has not previously been reported in the searched literature. Fluctuation in profiles may reflect measurement variability and inconsistencies or daily autonomic fluctuation. This variability complicates diagnosis and monitoring, suggesting that single-point testing may underestimate disease burden. Future protocols should consider timing LT assessments at the time of day when the patient is most symptomatic and incorporate repeated measures to capture these fluctuations.

Although quantitative improvements were modest, the intervention was well tolerated, and no serious adverse events were attributable to the program. These findings support the feasibility of implementing structured conservative management strategies for patients with symptomatic dysautonomia if expectations are managed and targets are realistic and personalised. Although quantitative data was collected as part of this study, management should be guided by symptoms and help patients to focus on the bigger picture rather than become preoccupied with tracking their own physiological parameters.

This study is limited by its small sample size, which has been compounded by reduced LC referrals to the services. This study did not use a control group and relied on self-reported adherence and symptom reports, which may be subject to recall bias. However, patients incorporated the DMP into their daily lives, and the complexities of this and the fluctuations in their symptoms related to their conditions cannot be isolated, which makes the results relatable to other patients. More than 90% of the study population was female, and whilst this reflects the clinical epidemiology of these conditions, it does limit how findings can be applied to a heterogenous population. If a larger study population were used or if there was a higher proportion of males to females, then potential sex-related differences in autonomic dysfunction or treatment response may be possible to identify. Using remote 10 min LT also introduces variability, with potential systematic and random error, which may influence HR and BP outcomes. In addition, the reported statistical significance may represent type I error, for which adjustment with Bonferroni correction would be important in a larger scale study appropriately powered to examine efficacy of the interventions. Statistical analysis could be strengthened with more thorough utilisation of methods such as repeated-measures ANOVA or mixed-effects models to account for within-subject variability over time. Recruitment challenges reflect the severity of symptoms and the high commitment required, potentially introducing selection bias. Furthermore, the short duration of implementation and assessment may not capture the full therapeutic potential of these interventions, which could justify adequately powered trials to confirm efficacy. Additionally, qualitative studies may elucidate patient perspectives on intervention burden and acceptability, informing refinements to protocol design.

## 5. Conclusions

To our knowledge, this study is the first to investigate the efficacy of a DMP comprising commonly used non-pharmacological treatments. The DMP was associated with a modest, statistically significant improvement in autonomic symptom burden (COMPASS-31) and a small, statistically significant reduction in postural heart rate, but no statistically significant improvements in blood pressure parameters or YRS scores. The non-pharmacological interventions were safe in this cohort of patients with multiple comorbidities. Patients could not fully implement the intervention targets during the 8-week study timescale, which limits our understanding of the actual efficacy, although this indicates that these commonly prescribed targets are difficult to achieve. The dysautonomia profiles of PoTS and OH seem to fluctuate considerably in terms of the 10 min LT findings.

The recommendations from this study are that clinicians need to set realistic expectations for the efficacy of non-pharmacological treatments with patients. There is a need for a larger-scale controlled study to provide a longer period of assessment, investigate a dose-response effect, and include pharmacological interventions. It will be difficult to estimate the efficacy of fully implementing treatment targets, as compliance is likely to be a limiting factor and results may be confounded by structured engagement with clinicians. A larger-scale study is needed to investigate whether the 10 min LT states in PoTS and OH constitute a single continuum or are distinct, as currently believed. The non-pharmacological interventions, however, remain the same regardless of the dysautonomia state (PoTS or OH) on the 10 min LT.

## Figures and Tables

**Table 1:** Baseline characteristics.

	Recruited DMP Patients *n* = 22	Completed DMP Patients *n* = 16
Demographics		
Age, years (range)	39 (17.25–63.2)	40.5 (18.8–63.2)
White British	18	14
Mixed	2	1
Arab	1	
White other	1	1
Female	20	15
Single	5	3
Married/in relationship	17	13
Professional occupation	7	6
Unemployed	6	3
Student	3	2
Associate professional	2	1
Caring role	2	2
Administrative role	1	1
Retired	1	1
Current smoker	2	2
Body mass index, kg/m^2^ (range)	29.2 (17.7–47.9)	28.3 (17.7–47.9)
Diagnosis		
ME/CFS	14	11
LC	8	5
Duration of dysautonomia symptoms, years (range)	7 (0.6–30)	8.3 (0.6–30)
Profile on baseline LT		
PoTS	20	15
OH	2	1

**Table 2:** Comorbidities disclosed by the recruited patients.

Comorbidities	Recruited DMP Patients *n* = 22	Completed DMP Patients *n* = 16
Fibromyalgia	10	7
Asthma	7	7
EDS/hypermobility syndrome	6	6
Depression and anxiety	4	4
Chronic pain	4	1
Migraines	4	4
Reflux	4	3
IBS	3	2
Raynaud’s	2	2
ADHD	2	2
Autism	2	2
Other conditions experienced with a frequency of one:	Type 2 diabetes, Hypertension, Fatty liver, Epilepsy, Overactive bladder, Endometriosis, Sleep apnoea, Hyperhidrosis, Restless legs, Vertigo	

**Table 3:** Medication taken by patients on the DMP.

	Recruited DMP Patients *n* = 22	Completed DMP Patients *n* = 16
Duloxetine	2	2
Gabapentin	5	4
Pregabalin	3	2
Amitriptyline	2	2
Paracetamol	4	4
Ibuprofen	2	2
Lansoprazole	2	2
Omeprazole	4	3
Symbicort inhaler	2	2
Sertraline	2	2
Fluoxetine	2	2
Mirtazapine	2	2
Fexofenadine	3	2
Desogestrel	5	4
Hormone replacement therapy	3	2
Other medications taken with a frequency of one:	Tramadol, nefopam, buscopan, clonidine, baclofen, montelukast, fostair inhaler, budesonide inhaler, salbutamol inhaler, metformin, gliclazide, semaglutide, empagliflozin, aspirin, ramipril, amlodipine, nifedipine, fludrocortisone, atorvastatin, methylphenidate, lamotrigine, escitalopram, citalopram, sumatriptan, propranolol (prn for anxiety), zolpidem, loratadine, cetirizine, ketotifen, mometasone nasal spray, clinitas carbomer eye gel, microgynon, codeine, naproxen, tranexamic acid, movicol, loperamide, colesevelam, mounjaro, famotidine	

**Table 4:** Outcomes during the DMP (* statistical significance).

	Baseline Score	Post-DMP Score	Change	*p* Value
COMPASS-31 YRS (SS score) YRS (FD score)	44.99	37.24	7.74 (improvement)	0.045 *
	19.13	17.13	2.0 (improvement)	0.16
	9.88	8.81	1.06 (improvement)	0.22
HR (2-week average) Systolic BP (2-week average)	33.81	28.93	4.88 (improvement)	0.03 *
	−5.09	−3.34	1.75 (improvement)	0.40
Diastolic BP (2-week average)	3.69	1.48	2.20 (deterioration)	0.13

**Table 5:** Variation in dysautonomia profile over 8-week serial NASA lean tests.

	LC	ME/CFS
PoTS only		4
PoTS or OH	1	4
PoTS or borderline	1	3
PoTS or OH or borderline	3	

## Data Availability

Data is available by contacting the corresponding author.

## Supplementary Materials

The following supporting information can be downloaded at: https://www.mdpi.com/article/10.3390/jcm15072510/s1, COMPASS-31 questionnaire, YRS questionnaire, 10 min LT template.

## Abbreviations

The following abbreviations are used in this manuscript:

DMP	Dysautonomia management protocol
ME/CFS	Myalgic encephalomyelitis/chronic fatigue syndrome
LC	Long COVID
OH	Orthostatic hypotension
PoTS	Postural orthostatic tachycardia syndrome
LT	Lean test
COMPASS-31	Composite Autonomic Symptom Score questionnaire
YRS	Yorkshire Rehabilitation Scale
PESE	Post exertional symptom exacerbation
UK	United Kingdom
NHS	National Health Service
BP	Blood pressure
HR	Heart rate
BMI	Body mass index
EDS	Ehlers–Danlos syndrome
