# Supplementary material for: Testing a Personalised Dysautonomia Management Protocol in Patients with Orthostatic Intolerance and a Diagnosis of Myalgic Encephalomyelitis/Chronic Fatigue Syndrome or Long COVID
Source: J Clin Med. 2026 Mar 25;15(7):2510. doi: 10.3390/jcm15072510 (PMC13072946; doi:10.3390/jcm15072510)
Supplement: Supplementary file 1 [file jcm-15-02510-s001.zip › jcm-4175851-supplementary.pdf]

Supplementary materials

1. COMPASS-31 questionnaire
2. YRS questionnaire
3. 10-min LT template

1. The Composite Autonomic Symptom Score (COMPASS-31)

1. In the past week, have you ever felt faint, dizzy, "goofy", or had difficulty thinking soon after standing up from a sitting or lying position?

1. Yes
2. No (if you marked No, please skip to question 5)

2. When standing up, how frequently do you get these feelings or symptoms?

1. Rarely
2. Occasionally
3. Frequently
4. Almost Always

3. How would you rate the severity of these feelings or symptoms?

1. Mild
2. Moderate
3. Severe

4. In the past week, have these feelings or symptoms that you have experienced:

1. Gotten much worse
2. Gotten somewhat worse
3. Stayed about the same
4. Gotten somewhat better
5. Gotten much better
6. Completely gone

5. In the past week, have you ever noticed color changes in your skin, such as red, white, or purple?

1. Yes
2. No (if you marked No, please skip to question 8)

6. What parts of your body are affected by these color changes? (Check all that apply)

1. Hands
2. Feet

7. Are these changes in your skin color:

1. Getting much worse
2. Getting somewhat worse
3. Staying about the same
4. Getting somewhat better
5. Getting much better
6. Completely gone

8. In the past 5 weeks, what changes, if any, have occurred in your general body sweating?

1. I sweat much more than I used to
2. I sweat somewhat more than I used to
3. I haven't noticed any changes in my sweating
4. I sweat somewhat less than I used to
5. I sweat much less than I used to

9. Do your eyes feel excessively dry?

1. Yes
2. No

10. Does your mouth feel excessively dry?

1. Yes
2. No

11. For the symptom of dry eyes or dry mouth that you have had for the longest period of time, is this symptom:

1. I have not had any of these symptoms
2. Getting much worse
3. Getting somewhat worse
4. Staying about the same
5. Getting somewhat better
6. Getting much better
7. Completely gone

12. In the past week, have you noticed any changes in how quickly you get full when eating a meal?

1. I get full a lot more quickly now than I used to
2. I get full more quickly now than I used to
3. I haven't noticed any change
4. I get full less quickly now than I used to
5. I get full a lot less quickly now than I used to

13. In the past week, have you felt excessively full or persistently full (bloated feeling) after a meal?

1. Never
2. Sometimes
3. A lot of the time

14. In the past week, have you vomited after a meal?

1. Never
2. Sometimes
3. A lot of the time

15. In the past week, have you had a cramping or colicky abdominal pain?

1. Never
2. Sometimes
3. A lot of the time

16. In the past week, have you had any bouts of diarrhea?

1. Yes
2. No (if you marked No, please skip to question 20)

17. How frequently does this occur?

1. Rarely
2. Occasionally
3. Frequently ____________ times per week
4. Constantly

18. How severe are these bouts of diarrhea?

1. Mild
2. Moderate
3. Severe

19. Are your bouts of diarrhea getting:

1. Much worse
2. Somewhat worse
3. Staying the same
4. Somewhat better
5. Much better
6. Completely gone

20. In the past week, have you been constipated?

1. Yes
2. No (if you marked No, please skip to question 24)

21. How frequently are you constipated?

1. Rarely
2. Occasionally
3. Frequently ____________ times per week
4. Constantly

22. How severe are these episodes of constipation?

1. Mild
2. Moderate
3. Severe

23. Is your constipation getting:

1. Much worse
2. Somewhat worse
3. Staying the same
4. Somewhat better
5. Much better
6. Completely gone

24. In the past week, have you ever lost control of your bladder function?

1. Never
2. Occasionally
3. Frequently ____________ times per week
4. Constantly

25. In the past week, have you had difficulty passing urine?

1. Never
2. Occasionally
3. Frequently ____________ times per week
4. Constantly

26. In the past week, have you had trouble completely emptying your bladder?

1. Never
2. Occasionally
3. Frequently ____________ times per week
4. Constantly

27. In the past week, without sunglasses or tinted glasses, has bright light bothered your eyes?

1. Never (if you marked Never, please skip to question 29)
2. Occasionally
3. Frequently
4. Constantly

28. How severe is this sensitivity to bright light?

1. Mild
2. Moderate
3. Severe

29. In the past week, have you had trouble focusing your eyes?

1. Never (if you marked Never, please skip to question 31)
2. Occasionally
3. Frequently
4. Constantly

30. How severe is this focusing problem?

1. Mild
2. Moderate
3. Severe

31. Is the most troublesome symptom with your eyes (i.e. sensitivity to bright light or trouble focusing) getting:

1. I have not had any of these symptoms
2. Much worse
3. Somewhat worse
4. Staying about the same
5. Somewhat better
6. Much better
7. Completely gone

2. Yorkshire Rehabilitation Scale (YRS)

The purpose of this questionnaire is to find out more about your current symptoms related to your health condition. This information can be used to monitor your symptoms, inform decisions to try and improve them, and assess the response to different interventions.

You will be asked to rate the severity of each problem on a scale of 0-3 now, where "Now" refers to how you feel now / this week (the last 7 days):

0 = None; no problem
1 = Mild problem; does not affect daily life
2 = Moderate problem; affects daily life to a certain extent
3 = Severe problem; affects all aspects of daily life; life-disturbing

Please answer each question to the best of your knowledge. This questionnaire will take around 15 minutes. If there are any topics you don't want to talk about you can choose not to respond.

Symptom Severity

1. Breathlessness	At rest	**0** ☐ **1** ☐ **2** ☐ **3** ☐
	Changing position e.g. from lying to sitting or sitting to lying	**0** ☐ **1** ☐ **2** ☐ **3** ☐
	On dressing yourself	**0** ☐ **1** ☐ **2** ☐ **3** ☐
	On walking up a flight of stairs	**0** ☐ **1** ☐ **2** ☐ **3** ☐
2. Cough/ throat sensitivity/ voice change	Cough/ throat sensitivity	**0** ☐ **1** ☐ **2** ☐ **3** ☐
	Change of voice	**0** ☐ **1** ☐ **2** ☐ **3** ☐
3. Fatigue (tiredness not improved by rest)	Fatigue levels in your usual activities	**0** ☐ **1** ☐ **2** ☐ **3** ☐
4. Smell/taste	Altered smell	**0** ☐ **1** ☐ **2** ☐ **3** ☐
	Altered taste	**0** ☐ **1** ☐ **2** ☐ **3** ☐
5. Pain/discomfort	Chest pain	**0** ☐ **1** ☐ **2** ☐ **3** ☐
	Joint pain	**0** ☐ **1** ☐ **2** ☐ **3** ☐
	Muscle pain	**0** ☐ **1** ☐ **2** ☐ **3** ☐
	Headache	**0** ☐ **1** ☐ **2** ☐ **3** ☐

	Abdominal pain	**0** ☐ **1** ☐ **2** ☐ **3** ☐
6. Cognition	Problems with concentration	**0** ☐ **1** ☐ **2** ☐ **3** ☐
	Problems with memory	**0** ☐ **1** ☐ **2** ☐ **3** ☐
	Problems with planning	**0** ☐ **1** ☐ **2** ☐ **3** ☐
7. Palpitations/ dizziness	Palpitations in certain positions, activity or at rest	**0** ☐ **1** ☐ **2** ☐ **3** ☐
	Dizziness in certain positions, activity or at rest	**0** ☐ **1** ☐ **2** ☐ **3** ☐
8. Post-exertional malaise (worsening of symptoms)	Crashing or relapse hours or days after physical, cognitive or emotional exertion	**0** ☐ **1** ☐ **2** ☐ **3** ☐
9. Anxiety/ mood	Feeling anxious	**0** ☐ **1** ☐ **2** ☐ **3** ☐
	Feeling depressed	**0** ☐ **1** ☐ **2** ☐ **3** ☐
	Having unwanted memories of your illness	**0** ☐ **1** ☐ **2** ☐ **3** ☐
	Having unpleasant dreams about your illness	**0** ☐ **1** ☐ **2** ☐ **3** ☐
	Trying to avoid thoughts or feelings about your illness	**0** ☐ **1** ☐ **2** ☐ **3** ☐
10. Sleep	Sleep problems, such as difficulty falling asleep, staying asleep or oversleeping	**0** ☐ **1** ☐ **2** ☐ **3** ☐

Functional Ability

11. Communication	Difficulty with communication/word finding difficulty/understanding others	**0** ☐ **1** ☐ **2** ☐ **3** ☐
12. Walking or moving around	Difficulties with walking or moving around	**0** ☐ **1** ☐ **2** ☐ **3** ☐
13. Personal care	Difficulties with personal tasks such as using the toilet or getting washed and dressed	**0** ☐ **1** ☐ **2** ☐ **3** ☐

14. Other activities of Daily Living	Difficulty doing wider activities, such as household work, leisure/sporting activities, paid/unpaid work, study, or shopping	**0** ☐ **1** ☐ **2** ☐ **3** ☐
15. Social role	Problems with socialising/interacting with friends or caring for dependants	**0** ☐ **1** ☐ **2** ☐ **3** ☐

Other Symptoms

Please select any of the following symptoms you have experienced since your illness in the last 7 days. Please also select any previous problems that have worsened for you following your illness.

☐ Fever

☐ Skin rash/ discolouration of skin

☐ New allergy such as medication, food etc

☐ Hair loss

☐ Skin sensation (numbness/tingling/itching/nerve pain)

☐ Dry eyes/ redness of eyes

☐ Swelling of feet/ swelling of hands

☐ Easy bruising/ bleeding

☐ Visual changes

☐ Difficulty swallowing solids

☐ Difficulty swallowing liquids

☐ Balance problems or falls

☐ Weakness or movement problems or coordination problems in limbs

☐ Tinnitus

☐ Nausea

☐ Dry mouth/mouth ulcers

☐ Acid Reflux/heartburn

☐ Change in appetite

☐ Unintentional weight loss

☐ Unintentional weight gain

☐ Bladder frequency,urgency or incontinence

☐ Constipation, diarrhoea or bowel incontinence

☐ Change in menstrual cycles or flow

☐ Waking up at night gasping for air (also called sleep apnea)

☐ Thoughts about harming yourself

If you are thinking about self-harm please speak to your GP or call the mental health crisis team on 0800 183 1485 (open 24/7, every day) or call 999.

Other symptoms:

Overall Health

How good or bad is your health overall in the last 7 days? NB: PLEASE NOTE THAT THIS QUESTION IS SCORED IN THE OPPOSITE DIRECTION TO THE REST OF THE QUESTIONS IN THIS QUESTIONNAIRE.

For this question, a score of 10 means the BEST health you can imagine. 0 means the WORST health you can imagine.

WORST HEALTH 0 ☐ 1 ☐ 2 ☐ 3 ☐ 4 ☐ 5 ☐ 6 ☐ 7 ☐ 8 ☐ 9 ☐ 10 ☐ BEST HEALTH

Employment

Occupation: ____

Have your ongoing symptoms affected your work?

☐ No change

☐ On reduced working hours

☐ On sickness leave

☐ Changes made to role/ working arrangements (such as working from home or lighter duties)

☐ Had to retire/ change job

☐ Lost job

Any other comments/concerns:

Partner/Family/Carer Perspective

This is space for your partner, family or carer to add anything from their perspective:

Many thanks for completing this questionnaire; this will allow us to review your progress.

3. 10-min LT template

**10-min Lean Test data sheet**			
When recording your blood pressure, please ensure that you:<br>• use an upper arm cuff blood pressure monitor.<br>• use the same arm for every recording.<br>• have your arm supported at the level of your heart in each position.<br>• are relaxed with your hand not clenched and that you are not talking.			
**Step 1:** Lay down and measure your heart rate and blood pressure once after 2-5 min of lying down and being comfortable			
	**Heart Rate** (e.g., 76)	**Blood Pressure** (e.g., 120/80)	**Symptoms** (e.g., felt dizzy, headache, etc.)
**3 minutes of lying down**			
**5 minutes of lying down**			
**Step 2:** Stand up without leaning on anything or leaning with just your shoulder blades against a wall.<br>Then, measure heart rate and blood pressure every 1-2 minutes whilst standing, for 10 minutes.			
	**Heart Rate**	**Blood Pressure**	**Symptoms**
**On standing**			
**1 minutes after standing**			
**2 minutes after standing**			
**3 minutes after standing**			
**4 minutes after standing**			
**5 minutes after standing**			
**6 minutes after standing**			
**7 minutes after standing**			
**8 minutes after standing**			
**9 minutes after standing**			
**10 minutes after standing**			
